# Supplementary material for: High Mycobacterium bovis Exposure but Low IGRA Positivity in UK Farm Workers
Source: Zoonoses Public Health. 2025 Feb 25;72(4):369–78. doi: 10.1111/zph.13214 (PMC12016005; doi:10.1111/zph.13214)
Supplement: Supplementary file 1 — Data S1: Questionnaire. [file ZPH-72-369-s001.docx]

**Supplementary information**

**High *Mycobacterium bovis* exposure but low IGRA positivity in UK farm workers**

**Authors**

Amy Thomas^1^ ([amyc.thomas@bristol.ac.uk)](mailto:amyc.thomas@bristol.ac.uk)1)

Alice Halliday^2^ ([alice.halliday@bristol.ac.uk)](mailto:alice.halliday@bristol.ac.uk)2)

Genevieve Clapp^1^ ([pu18699@bristol.ac.uk)](mailto:pu18699@bristol.ac.uk)1)

Ross Symonds^1^ ([zooTB-study@bristol.ac.uk)](mailto:zooTB-study@bristol.ac.uk)3)

Noreen Hopewell-Kelly^3^ ([hopewell-kellyn2@cardiff.ac.uk)](mailto:hopewell-kellyn2@cardiff.ac.uk)4)

Carmel McGrath^4,5,6^ ([carmel.mcgrath@uwe.ac.uk)](mailto:carmel.mcgrath@uwe.ac.uk)5,6,7)

Lucy Wheeler^7^ ([lucy.wheeler@nbt.nhs.uk)](mailto:lucy.wheeler@nbt.nhs.uk)8)

Anna Dacey^7^ ([Anna.Dacey@nbt.nhs.uk)](mailto:Anna.Dacey@nbt.nhs.uk)8)

Nigel Noel^7^ ([Nigel.Noel@nbt.nhs.uk)](mailto:Nigel.Noel@nbt.nhs.uk)8)

Andrea Turner^8^ ([andrea.turner@bristol.ac.uk)](mailto:andrea.turner@bristol.ac.uk)9)

Isabel Oliver^4,9^ ([isabel.oliver@phe.gov.uk)](mailto:isabel.oliver@phe.gov.uk)5,10)

James Wood^10^ ([jlnw2@cam.ac.uk)](mailto:jlnw2@cam.ac.uk)11)

Ed Moran^11^ ([ed.moran@nbt.nhs.uk)](mailto:ed.moran@nbt.nhs.uk)12)

Paul Virgo^7^ ([pfvir@aol.com](mailto:pfvir@aol.com))

James Tiller^13^ ([james.tiller@apha.gov.uk](mailto:james.tiller@apha.gov.uk))

Paul Upton^13^ ([Paul.Upton@apha.gov.uk](mailto:Paul.Upton@apha.gov.uk) )

Andrew Mitchell^13^ ([Andrew.Mitchell@apha.gov.uk](mailto:Andrew.Mitchell@apha.gov.uk))

Anu Goenka^2,12^ ([anu.goenka@bristol.ac.uk)](mailto:anu.goenka@bristol.ac.uk)2,13)

Ellen Brooks-Pollock^1,4^ ([ellen.brooks-pollock@bristol.ac.uk)](mailto:ellen.brooks-pollock@bristol.ac.uk)1,5*)

**Affiliations**

^1^Population Health Sciences, Bristol Medical School, University of Bristol, Bristol BS8 2BN, UK.

^2^Bristol Vaccine Centre, School of Cellular and Molecular Medicine, University of Bristol, UK

^3^ Division of Population Medicine, School of Medicine, Cardiff University, Heath Park Cardiff, CF14 4YS, UK

^4^NIHR Health Protection Research Unit in Behavioural Science and Evaluation, Population Health Sciences, Bristol Medical School, University of Bristol, Bristol, UK.

^5^The National Institute for Health and Care Research Applied Research Collaboration West (NIHR ARC West) at University Hospitals Bristol and Weston NHS Foundation Trust, Bristol, UK

^6^ Faculty of Health and Applied Sciences, School of Health and Social Wellbeing, University of West England, Bristol, UK.

^7^North Bristol NHS Trust, Bristol, UK

^8^University of Bristol Veterinary School, Langford, BS40 5DU, UK.

^9^UK Health Security Agency, London, UK

^10^Cambridge Veterinary School, University of Cambridge, Cambridge, CB3 0ES, UK.

^11^Department of Infectious Disease, North Bristol NHS Trust, Bristol, United Kingdom

^12^Department of Paediatric Immunology and Infectious Diseases, Bristol Royal Hospital for Children, Bristol, UK.

^13^ Animal and Plant Health Agency, New Haw, Addlestone, Surrey, KT15 3NB, UK

*Author for correspondence: Ellen Brooks-Pollock, [Ellen.Brooks-Pollock@bristol.ac.uk](mailto:Ellen.Brooks-Pollock@bristol.ac.uk).

**ZooTB study questionnaire**
