# Supplementary material for: High Mycobacterium bovis Exposure but Low IGRA Positivity in UK Farm Workers
Source: Zoonoses Public Health. 2025 Feb 25;72(4):369–78. doi: 10.1111/zph.13214 (PMC12016005; doi:10.1111/zph.13214)
Supplement: Supplementary file 2 — Data S2. Graphical Abstract. [file ZPH-72-369-s002.pdf]

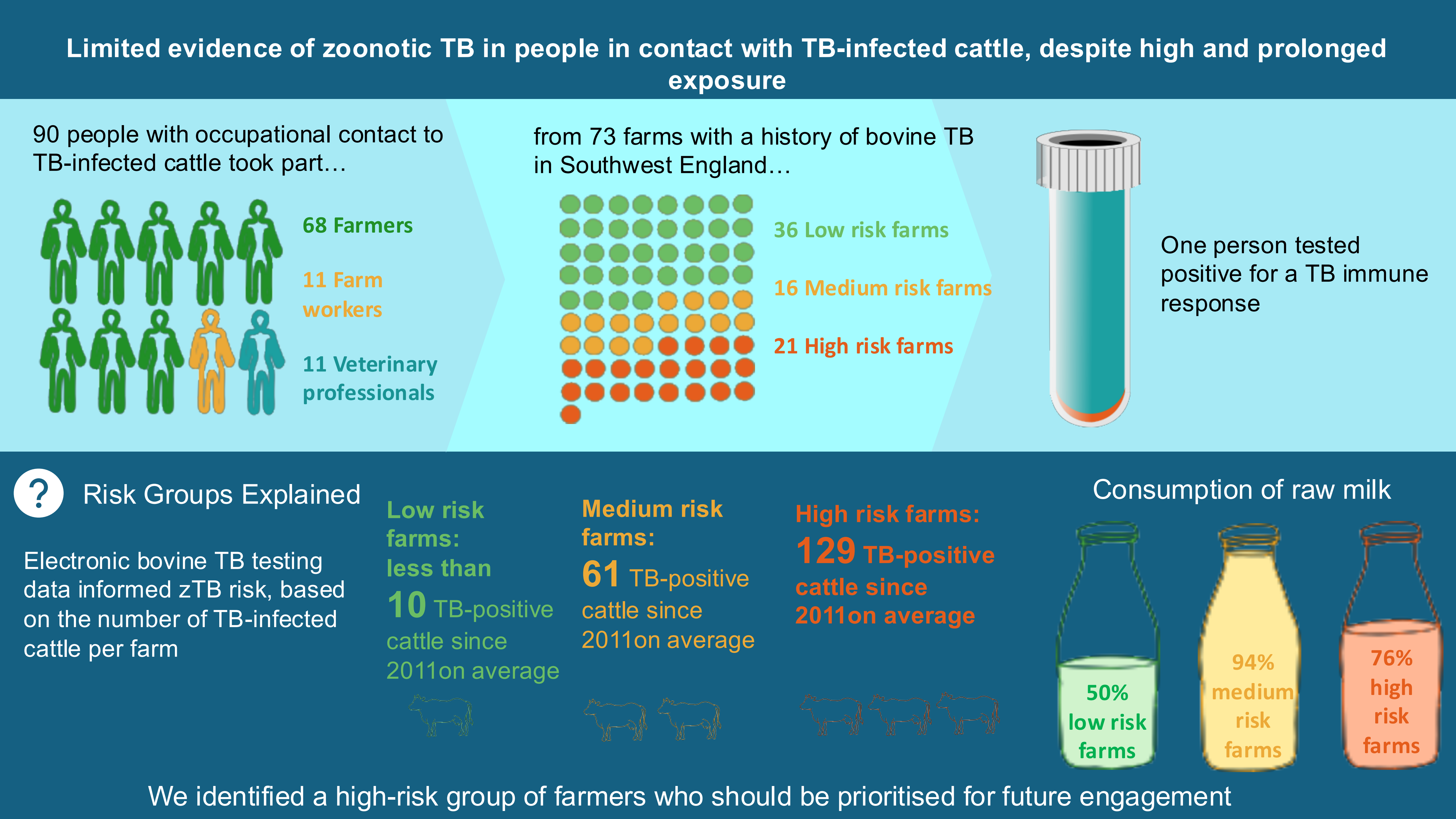
Limited evidence of zoonotic TB in people in contact with TB-infected cattle, despite high and prolonged exposure
90 people with occupational contact to TB-infected cattle took part…
68 Farmers
11 Farm workers
11 Veterinary professionals
from 73 farms with a history of bovine TB in Southwest England…
36 Low risk farms
16 Medium risk farms
21 High risk farms
One person tested positive for a TB immune response
Risk Groups Explained
Electronic bovine TB testing data informed zTB risk, based on the number of TB-infected cattle per farm
Low risk farms: less than 10 TB-positive cattle since 2011on average
Medium risk farms: 61 TB-positive cattle since 2011on average
High risk farms: 129 TB-positive cattle since 2011on average
Consumption of raw milk
50% low risk farms
94% medium risk farms
76% high risk farms
We identified a high-risk group of farmers who should be prioritised for future engagement
